# Circulating anions usually associated with the Krebs cycle in patients with metabolic acidosis

**DOI:** 10.1186/cc3806

**Published:** 2005-09-13

**Authors:** Lui G Forni, William McKinnon, Gwyn A Lord, David F Treacher, Jean-Marie R Peron, Philip J Hilton

**Affiliations:** 1 Consultant Physician & Intensivist, Department of Critical Care, Worthing Hospital, Worthing, West Sussex, UK; 2 Research Fellow, Renal Laboratory, St Thomas' Hospital, London, UK; 3 MRC Scientist, MRC Toxicology Unit, Birkbeck College, London, UK; 4 Consultant Physician & Intensivist, Renal Laboratory, St Thomas' Hospital, London, UK; 5 Research Fellow, Department of Chemistry, Kingston University, Surrey, UK; 6 Consultant Physician & Research Director, Renal Laboratory, St Thomas' Hospital, London, UK

## Abstract

**Introduction:**

Acute metabolic acidosis of non-renal origin is usually a result of either lactic or ketoacidosis, both of which are associated with a high anion gap. There is increasing recognition, however, of a group of acidotic patients who have a large anion gap that is not explained by either keto- or lactic acidosis nor, in most cases, is inappropriate fluid resuscitation or ingestion of exogenous agents the cause.

**Methods:**

Plasma ultrafiltrate from patients with diabetic ketoacidosis, lactic acidosis, acidosis of unknown cause, normal anion gap metabolic acidosis, or acidosis as a result of base loss were examined enzymatically for the presence of low molecular weight anions including citrate, isocitrate, α-ketoglutarate, succinate, malate and d-lactate. The results obtained from the study groups were compared with those obtained from control plasma from normal volunteers.

**Results:**

In five patients with lactic acidosis, a significant increase in isocitrate (0.71 ± 0.35 mEq l^-1^), α-ketoglutarate (0.55 ± 0.35 mEq l^-1^), malate (0.59 ± 0.27 mEq l^-1^), and d-lactate (0.40 ± 0.51 mEq l^-1^) was observed. In 13 patients with diabetic ketoacidosis, significant increases in isocitrate (0.42 ± 0.35 mEq l^-1^), α-ketoglutarate (0.41 ± 0.16 mEq l^-1^), malate (0.23 ± 0.18 mEq l^-1^) and d-lactate (0.16 ± 0.07 mEq l^-1^) were seen. Neither citrate nor succinate levels were increased. Similar findings were also observed in a further five patients with high anion gap acidosis of unknown origin with increases in isocitrate (0.95 ± 0.88 mEq l^-1^), α-ketoglutarate (0.65 ± 0.20 mEq l^-1^), succinate (0.34 ± 0.13 mEq l^-1^), malate (0.49 ± 0.19 mEq l^-1^) and d-lactate (0.18 ± 0.14 mEq l^-1^) being observed but not in citrate concentration. In five patients with a normal anion gap acidosis, no increases were observed except a modest rise in d-lactate (0.17 ± 0.14 mEq l^-1^).

**Conclusion:**

The levels of certain low molecular weight anions usually associated with intermediary metabolism were found to be significantly elevated in the plasma ultrafiltrate obtained from patients with metabolic acidosis. Our results suggest that these hitherto unmeasured anions may significantly contribute to the generation of the anion gap in patients with lactic acidosis and acidosis of unknown aetiology and may be underestimated in diabetic ketoacidosis. These anions are not significantly elevated in patients with normal anion gap acidosis.

## Introduction

Metabolic acidosis is a common presentation in acute medicine and in only a minority of cases can renal failure alone be considered the sole cause of the acidosis. Diabetic ketoacidosis (DKA) and 'classic' lactic acidosis (taken as a blood lactate in excess of 5 mmol l^-1^) account for most of the remaining cases [1,2]. Amongst clinicians there is an increasing awareness that there is often an important discrepancy between the measured acidosis and the associated base deficit, suggesting that other anions must contribute to the generation of the anion gap [3,4]. Further evidence to support the hypothesis of additional 'missing anions' is supplied by observations on patients receiving bicarbonate-buffered haemofiltration for treatment of lactic acidosis where it is possible to calculate the net rates of lactate removal and bicarbonate donation by the haemofilter [5]. In patients whose blood pH is slowly falling (thereby excluding any possible confounding effect of other blood buffers), it is not uncommon for the rate of bicarbonate donation to exceed the rate of lactate removal by as much as twofold (PJ Hilton, unpublished observations). This implies that not only lactic acid, but other 'unknown' acids are being neutralised by the bicarbonate in the haemofiltration replacement fluid.

Preliminary experiments performed in this laboratory with high performance liquid chromatography coupled to mass spectrometry on plasma ultrafiltrate obtained from patients with metabolic acidosis had suggested that anions principally associated with the Krebs tricarboxylic acid cycle were significantly elevated in patients with unexplained metabolic acidosis. We undertook an enzymatic study of anions of the Krebs cycle in patients with metabolic acidosis whose standard base deficit was 8 mmol l^-1 ^or greater. The samples were obtained from consecutive patients who fulfilled these requirements except those with a normal anion gap, which were collected subsequently. The results obtained from the patient samples were compared to control values obtained from healthy volunteers.

## Methods

This study was approved by the Ethics Committee of Guy's and St Thomas' National Health Service Trust (reference number EC03/104). Prior to the sample being taken, informed consent was obtained from the subject or, where this was not possible, their next of kin. Patient studies were undertaken on 15 ml of arterial blood taken from arterial cannulae in patients with metabolic acidosis whose standard base deficit was 8 mmol l^-1 ^or greater. The arterial blood was drawn into a non-heparinised syringe before being rapidly transferred into SST II (KODAK) Vaccutainers (BD Vaccutainer Systems Ltd, Plymouth, UK). Control samples were obtained from venous blood of laboratory workers and treated in the same manner as that obtained from patients. Arterial blood gas levels were not measured as it was felt that arterial puncture was inappropriate.

Once obtained, the sample was chilled and rapidly transported to the laboratory. The plasma was isolated by centrifugation of the Vaccutainers (1,500 g) at 4°C for 10 minutes. The plasma was transferred to an Amicon 30,000 Da cutoff filter (Millipore, Watford, Herts, UK) where centrifugation at 1,560 g for 15 minutes produced ultrafiltrate. The generated ultrafiltrate was either immediately analysed or stored at -20°C for analysis within 24 h. Previous work had highlighted the need for rapid assay of the samples due to an observed rapid decrease in concentrations of the measured anions within plasma.

The concentration of anions in the ultrafiltrate was determined by enzymatic assay with reference to internal standards. The plasma ultrafiltrate concentrations of citrate, succinate, malate, d-lactate and l-lactate anions were estimated using commercially available kits (Roche, Glasgow, UK). The levels of isocitrate and α-ketoglutarate were measured using enzyme assays developed by ourselves using isocitrate dehydrogenase and α-ketoglutarate dehydrogenase, respectively, and their associated co-factors (Sigma Chemicals, Poole, UK). All the enzymatic assays relied upon the interconversion of NAD^+ ^and NADH or NADP^+ ^and NADPH and used the change in absorbance due to the reduced co-enzyme at 340 nm. Oxaloacetate concentrations could not be accurately measured as a result of its short half-life (approximately 69 s) in aqueous systems at near physiological pH [6]. All data are presented as mean ± standard deviation. Unless stated otherwise, the data are normally distributed and statistical analysis was undertaken with an unpaired t-test. Where the data was not normally distributed a Mann-Whitney non-parametric test was applied. In both cases, significance was deemed to have been attained if *p *was ≤0.05

## Results

Samples were obtained from 28 patients in total and from 12 control subjects. Of the study patients, 13 had DKA at the time of blood sampling and 5 had a lactic acidosis (defined as a blood lactate concentration in excess of 5 mmol/l in the presence of acidosis). Five patients had a metabolic acidosis that could not be ascribed to either lactic acidosis, ketoacidosis or exogenous agents. A further five patients had an acidosis as a result of gastrointestinal or renal ion losses. In all cases, the patients were acidotic with an average pH of 7.18 (±0.11) and a base deficit of 13.4 (±4.7) mmol l^-1^.

**Figure 1:**
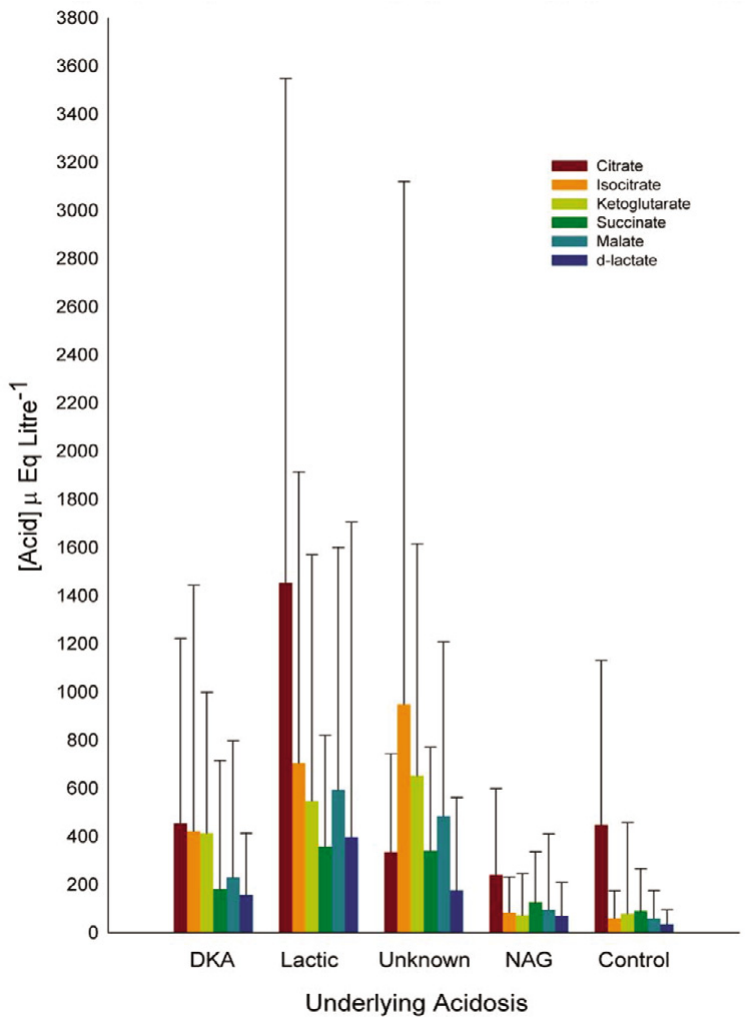
The concentration of various weak acids grouped by their underlying aetiology (mean ± upper range). DKA, diabetic ketoacidosis; NAG, normal anion gap.

The mean results obtained for the plasma ultrafiltrate concentration of citrate, succinate, malate, d-lactate, l-lactate, isocitrate and α-ketoglutarate are presented in Table 1. The mean results obtained from the data together with the maximal value for each anion measured in each type of acidosis are shown in Fig. 1.

Patients with DKA showed significant increases relative to the control values in isocitrate, α-ketoglutarate, malate and d-lactate levels. The concentrations of both citrate and succinate did not differ significantly from controls. Patients with lactic acidosis showed significant increases relative to the control values in citrate, isocitrate, α-ketoglutarate, succinate, malate and d-lactate levels. Patients with acidosis of unknown origin showed significant increases in the concentrations of isocitrate, α-ketoglutarate, succinate, malate and d-lactate. The level of citrate did not differ significantly from that of the controls. In those patients with a normal anion gap acidosis, the levels of citrate, isocitrate, α-ketoglutarate, succinate and malate did not differ significantly from the control values. The concentration of d-lactate was significantly raised compared to control values in this patient group.

## Discussion

The consequences of metabolic acidosis can be catastrophic and a considerable body of literature highlights the poor outlook in patients where lactic acid is the principal component of the acidaemia [7-9]. This increase in blood lactate concentration reflects either increased lactate production, reduced lactate metabolism or, more commonly, a combination of the two [10]. In patients with DKA, 3-hydroxybutyric acid and, to a lesser extent, acetoacetic acid play the major role in the generation of the anion gap. As outlined, however, in a third patient group, neither lactate nor 3-hydroxybutyrate is responsible for the elevated anion gap and the relevant anions responsible remain unknown [11]. In the fourth patient group, acidosis is generated as a result of uncontrolled electrolyte loss either from the kidney (renal tubular acidosis) or the gut.

We have shown that the plasma concentrations of acids usually associated with the Krebs tricarboxylic acid cycle are significantly increased in patients with lactic acidosis as well as those with 'unexplained acidosis' with normal or near normal blood lactate concentrations. In DKA, although the concentrations of these acids are less strikingly elevated, they are still abnormal in the majority of patients when compared to controls. They are not, however, significantly elevated in patients with normal anion gap acidosis secondary to excess base loss. The accumulation of such acids may contribute significantly to the production of the anion gap and account, in part, for the 'missing' anions in patients with certain forms of acidosis. Recent studies, in keeping with previous work, have demonstrated the predictive value of acid-base variables on outcome in the critically ill [12]. Furthermore, the calculation of unmeasured anions appears to be a better discriminator of outcome than lactate or base deficit [13].

With the partial exception of citrate and isocitrate (97% ionised at pH 7.0) the anions examined in this study are effectively fully ionised at the measured pH. Unlike lactate, not all the anions are monobasic, with tribasic acids (citric and isocitric) contributing three protons, whilst the dibasic acids (α-ketoglutaric, malic and succinic) add two protons to the solution on ionisation. Converting the concentrations of these observed anions to mEq l^-1 ^(Table 1) shows that, on average, the contribution to the observed anion gap due to such anions may be in excess of 3 mEq l^-1 ^and in some cases may be over 5 mEq l^-1^. Thus, the contribution of these anions to the generation of the anion gap is of much greater significance than is apparent from their molarity. The large standard deviation present in these samples probably reflects their heterogeneous nature and, in many ways, the range and maxima in each group are of as much interest as the means, demonstrating the extreme ranges that can be present in patients with metabolic acidosis (Fig. 1).

The greatest deviations in the level of measured plasma ultrafiltrate acids from that observed in the control group were seen in the patients within the lactic acidosis and 'unexplained acidosis' groups. In these two groups, the concentrations of most of the acids studied were present in the plasma ultrafiltrate at a concentration significantly higher than that observed in the normal control population. In those with DKA, four of the six acids measured were significantly elevated relative to the control values. Interestingly, in normal anion gap acidosis, only d-lactate was significantly increased relative to the control values.

It has been widely reported elsewhere that plasma citrate is not elevated in acidosis [4,11] and we observed this in all the groups of patients studied except those with lactic acidosis. In this group, significantly elevated levels of citrate were observed in comparison to the normal control values. This result may be unreliable as four of the patients in this group had received an infusion of heparin (containing sodium citrate as an anticoagulant) prior to the blood sample being obtained. Isocitrate concentrations were significantly elevated in patients with ketoacidosis, lactic acidosis and those whose acidosis was of unknown origin. Consequently, the citrate:isocitrate ratio is significantly reduced compared to control values and we are unable to advance a simple explanation as to why this ratio should be so low.

Although it seems reasonable to believe that the likely source for the generation of these observed anions is the mitochondria, we have no direct evidence for this and the results may indeed reflect compartmentalised oxidative and glycolytic energy production. Indeed, studies on mitochondria in isolated rat skeletal muscle have demonstrated that lactic acidosis has differential effects on actively phosphorylating and non-phosphorylating mitochondria, suggesting that the effect of acidaemia may depend on local physiological conditions [14]. The rate of oxygen delivery to respiring tissue may also play a role in generating intermediates of Krebs acids, with several authors suggesting that hypoxia can cause an increase in intermediates of the citric acid cycle [15-18], although in the patients examined in this study none were significantly hypoxic at the time the sample was taken. Furthermore, it seems unlikely that the acidaemia *per se *is responsible for the increased levels of Krebs intermediates within our patient population given the normal values found in the patients with normal anion gap acidosis and the lesser elevation seen in the patients with DKA. An alternative explanation may be the generation of intermediates from anaplerotic pathways of metabolism, which may reflect enhanced protein catabolism in these patients

Other authors have proposed alternative hypotheses to explain how the 'missing' ions in the elevated anion gap may be generated. These include the choice of fluid used for resuscitation, the effects of hypoproteinaemia or the presence of other metabolites [19-21], in addition to various 'physical chemical' approaches, including the calculation of the strong anion difference [22]. This approach, which enjoys some popularity at the present time, represents an alternative way to express the principles of acidosis. The 'strong ion difference' model can be thought of as a restatement of the older concept of buffer base and thus, predictably, must approach the corrected anion gap, although some of its stronger proponents suggest that it can explain the generation of acidosis *per se*. We prefer to adhere to the concept that acidosis can best be regarded as an excess of protons over those normally found in physiological states, caused either from the loss of base or excessive net proton production.

We have previously referred to the difficulties of measuring plasma oxaloacetate in view of its extremely short half-life in aqueous solution. Given the increases in plasma concentrations of the other components of the Krebs cycle and the known existence of cytosolic oxaloacetate, it seems likely that some oxaloacetate does enter the plasma of these acidotic patient groups along with the other acids described here. There, it would spontaneously decarboxylate to pyruvate and its presence might only be inferred as a small deviation of the pyruvate:lactate ratio from that predicted on the basis of pH alone.

The results obtained from this study suggest that the role of anions principally associated with the Krebs cycle may be greater than previously thought in the generation of the anion gap in 'classic' lactic acidosis. In addition, these anions appear to have a significant role in the generation of the anion gap in patients with acidosis of unknown cause.

## Conclusion

We have shown that the concentration of anions normally associated with the Krebs tricarboxylic acid cycle are elevated in appreciable quantities in patients with a metabolic acidosis. We propose that they may play a significant role in generating the anion gap. Further work in this laboratory is currently underway to explore the clinical implications of these findings.

## Key messages

• 	Low molecular weight anions usually associated with intermediary metabolism are significantly elevated in the plasma ultrafiltrate obtained from patients with metabolic acidosis.

• 	These anions may contribute significantly to the elevated anion gap observed in patients with metabolic acidosis, in particular those of unknown aetiology.

## Abbreviations

DKA = diabetic ketoacidosis.

## Competing interests

This work was supported by The Special Trustees for St Thomas' Hospital, London. The authors have no financial interests relevant to the results of this research, nor are there any other circumstances that could potentially provoke a conflict of interest.

## Authors' contributions

LGF conceived the study, participated in its design, collected patient samples and drafted the manuscript. WM developed and performed the enzyme assays, participated in the design of the study, performed the statistical analysis and helped to draft the manuscript. GAL participated in the design of the study, performed the initial mass spectrometry and helped in assay development. DFT collected patient samples and participated in study design. JMP helped in assay development and stability studies. PJH conceived the study, participated in its design, collected patient samples, helped in assay development and helped to draft the manuscript. All authors read and approved the final manuscript.

## Figures and Tables

**Table 1:** Concentrations of measured anions in plasma (μEq l^-1^)

Acid	Patient group													
	Diabetic ketoacidosis			Lactic acidosis			Unknown origin			Normal anion gap			Controls	

	Mean	SD	p	Mean	SD	p	Mean	SD	p	Mean	SD	p	Mean	SD

Citrate	454.02	194.11	ns	1,453.13	513.95	<0.01	335.63	69.82	ns^a^	239.1	105.1	ns	448.55	119.80
Isocitrate	421.93	352.56	0.02	704.6	347.6	<0.01	949.16	883.22	<0.01^a^	84.48	69.6	ns^a^	60.97	31.31
α-Ketoglutarate	413.41	158.48	<0.01^a^	547.72	344.98	<0.01^a^	651.51	203.06	<0.01^a^	72.97	67.34	ns^a^	79.17	106.74
Succinate	181.1	173.24	ns	358.27	112.49	<0.01^a^	340.04	128.74	0.02^a^	125.89	73.57	ns^a^	90.29	49.97
Malate	229.81	181.87	<0.01^a^	593.65	265.88	<0.01	485.16	189.67	<0.01^a^	95.07	117.59	ns	59.82	32.94
d-Lactate	157.34	67.85	<0.01	397.69	511.15	<0.01^a^	176.49	135.21	<0.01	69.27	55.39	<0.01	35.63	18.42

For each group, statistical analysis is presented relative to control value. ^a^Mann-Whitney non-parametric test. ns, not significant; SD, standard deviation.
